# Response process and test–retest reliability of the Context Assessment for Community Health tool in Vietnam

**DOI:** 10.3402/gha.v9.31572

**Published:** 2016-06-10

**Authors:** Duong M. Duc, Anna Bergström, Leif Eriksson, Katarina Selling, Bui Thi Thu Ha, Lars Wallin

**Affiliations:** 1 Faculty of Social Science – Behaviours and Health Education, Hanoi School of Public Health, Hanoi, Vietnam; 2 International Maternal and Child Health, Department of Women's and Children's Health, Uppsala University, Uppsala, Sweden; 3 Institute for Global Health, University College London, London, UK; 4 School of Education, Health and Social Studies, Dalarna University, Falun, Sweden; 5 Department of Neurobiology, Care Sciences and Society, Karolinska Institutet, Stockholm, Sweden

**Keywords:** knowledge translation, context assessment, response process, think-aloud interview, test–retest, validity, reliability, implementation science

## Abstract

**Background:**

The recently developed Context Assessment for Community Health (COACH) tool aims to measure aspects of the local healthcare context perceived to influence knowledge translation in low- and middle-income countries. The tool measures eight dimensions (*organizational resources, community engagement, monitoring services for action, sources of knowledge, commitment to work, work culture, leadership, and informal payment*) through 49 items.

**Objective:**

The study aimed to explore the understanding and stability of the COACH tool among health providers in Vietnam.

**Designs:**

To investigate the response process, think-aloud interviews were undertaken with five community health workers, six nurses and midwives, and five physicians. Identified problems were classified according to Conrad and Blair's taxonomy and grouped according to an estimation of the magnitude of the problem's effect on the response data. Further, the stability of the tool was examined using a test–retest survey among 77 respondents. The reliability was analyzed for items (intraclass correlation coefficient (ICC) and percent agreement) and dimensions (ICC and Bland–Altman plots).

**Results:**

In general, the think-aloud interviews revealed that the COACH tool was perceived as clear, well organized, and easy to answer. Most items were understood as intended. However, seven prominent problems in the items were identified and the content of three dimensions was perceived to be of a sensitive nature. In the test–retest survey, two-thirds of the items and seven of eight dimensions were found to have an ICC agreement ranging from moderate to substantial (0.5–0.7), demonstrating that the instrument has an acceptable level of stability.

**Conclusions:**

This study provides evidence that the Vietnamese translation of the COACH tool is generally perceived to be clear and easy to understand and has acceptable stability. There is, however, a need to rephrase and add generic examples to clarify some items and to further review items with low ICC.

## Introduction

Failure to implement evidence-based practices (EBPs) results in the provision of inefficient or even harmful healthcare (1, 2). Although a number of knowledge translation (KT) strategies exist, there is currently an uncertainty about which implementation strategies work where, for whom, and under which circumstances (3, 4). The World Health Organization has urged researchers, policymakers and health providers to focus on evaluating different types of KT strategies (5). Furthermore, the nature of the context in which evidence is implemented has been put forward as mediating the success or failure of implementation efforts (3, 4). Therefore, a better understanding of context prior to the implementation of EBPs could assist in adapting effective healthcare interventions in new settings (4, 6), inform the decision on which implementation strategy to use (4, 6), and advance the understanding of variations (7, 8).

The Promoting Action on Research Implementation in Health Services framework was developed by researchers in the Royal College of Nursing Institute in the United Kingdom in the 1990s and emerged from working with clinicians on improving clinical practice (9). The framework outlines three core elements for successful implementation of EBP: evidence, context, and facilitation (10, 11). *Context* is defined as ‘the environment or setting in which the proposed change is to be implemented’ (12), p. 150). The context element is proposed to comprise three sub-elements: culture, leadership, and evaluation (11). Based on these context sub-elements, four tools have been developed to generate evidence on the effect of context in relation to KT interventions (13–16). To our knowledge, only the recently launched Context Assessment for Community Health (COACH) tool aims to assess healthcare context in low- and middle-income countries (LMICs). The COACH tool covers eight dimensions of context perceived to be of importance for the implementation of EBPs: *organizational resources, community engagement, monitoring services for action, sources of knowledge, commitment to work, work culture, leadership, and informal payment* (Table 1). The dimensions are measured through 49 items, where respondents are asked to rate their level of agreement on a five-point Likert scale for all items except those in the *sources of knowledge* dimension. In this dimension, the respondents are instead asked to state how often they use particular sources of knowledge in a ‘normal’ month.

**Table 1:** Definitions of dimensions of the COACH tool

Dimension	Definition
Organizational resources	The availability of resources that allow an organization (unit) to adapt successfully to internal and external pressures
Community engagement	The mutual communication, deliberation, and activities that occur between community members and an organization (unit)
Monitoring services for action	The process of using locally derived data to assess performance and plan how to improve outcomes in an organization (unit)
Sources of knowledge	The availability and use of sources of knowledge in an organization (unit) to facilitate best practice
Commitment to work	The individual's identification with and involvement in a particular organization (unit)
Work culture	The way ‘we do things’ in an organization (unit), reflecting a supportive work culture
Leadership	The actions of a formal leader in an organization (unit) to influence change and excellence in practice achieved through clarity and engagement
Informal payment	Payments or benefits given to individual(s) in an organization (unit), which are made outside the officially accepted arrangements, to acquire an advantage or service

COACH, Context Assessment for Community Health.

The COACH tool has been found to have acceptable reliability and validity among physicians, nurses and midwives, and community health workers (CHWs) in Vietnam, Bangladesh, Uganda, South Africa, and Nicaragua (13). As with all new psychometric tools, however, there is a need to generate further evidence to establish reliability and validity in diverse samples and settings. Some variations of psychometric properties across health professional groups and countries were also identified in the development process (13), calling for further examination of the tool. We got the opportunity to conduct an extended examination of the reliability and validity of the COACH tool in Vietnam. Therefore, the current study aimed to explore the understanding of the Vietnamese translation of the COACH tool among health providers in Vietnam (response process) as well as to assess the stability of the tool over time (test–retest).

## Methods

### Study setting

The study was conducted in Quang Ninh Province, located in north-eastern Vietnam. Health services in Quang Ninh are provided from the grass-roots level to the provincial level (17). Primary healthcare services, including assistance with normal births and basic outpatient care, are delivered at the commune health centers (CHCs), whereas most emergency and inpatient care is managed at district- or provincial-level hospitals (18). For the outreach activities, CHWs (also referred to as *village health workers* in Vietnam) are part-time health workers providing preventive services and collecting routine health data at the village level (18). Clients can seek health services from any level of the healthcare system; however, higher-level facilities charge clients higher user fees than lower-level facilities (19).

### Data collection and analysis

#### Response process using think-aloud methodology

To better understand how respondents comprehend the items and the cognitive processes that contribute to the resulting response decision, we assessed the response process by applying think-aloud methodology (20). Considering that the COACH tool was developed to assess context as perceived by various types of healthcare professionals, we opted to include CHWs, nurses, midwives, and physicians. Although they have different tasks, we grouped nurses and midwives together because of the similarities in their roles and the number of training years (13). In November 2014, 16 think-aloud interviews were undertaken with respondents (five CHWs, six nurses and midwives, and five physicians) working in purposively sampled CHCs in a district with average socio-economic characteristics. As the think-aloud interview is quite time-consuming, we opted to only cover half of the COACH tool with each respondent. Seven participants (two CHWs, three nurses and midwives, and two physicians) were asked about the first three dimensions (*organizational resources, community engagement, and monitoring services for action*). The other eight participants (three CHWs, three nurses and midwives, and two physicians) answered the remaining five dimensions (*sources of knowledge*, *commitment to work, work culture, leadership, and informal payment*). One physician did, however, complete the full tool.

Following an introduction to the tool, each participant rated their level of agreement with the items; they were then asked to verbalize their thoughts and express comments about the instructions related to the assigned dimensions and for each item. The interviewer asked the participants for clarification in instances where they expressed having difficulties in understanding and/or challenges in rating their level of agreement with an item. Finally, the participants were asked to express their overall thoughts regarding the assigned dimensions. The think-aloud interviews were undertaken in Vietnamese and audio recorded. Each interview lasted about 30–45 min.

The first author listened carefully to the audio recordings, transcribed them, and analyzed the identified problems using Conrad and Blair's taxonomy (20), outlining five types of problems (lexical problems, inclusion/exclusion problems, temporal problems, logical problems, and computational problems). All identified problems were translated into English and classification of the types of problems was discussed. The identified problems were also grouped into two categories according to our estimation of the magnitude of the problem's effect on response data: prominent versus minor problems (Table 2).

**Table 2:** Types of problems and level of effect regarding identified problems of items in the COACH tool

Five types of problems in Conrad and Blair's taxonomy (20) *Lexical problems*: difficulties in understanding the meaning of a word or a phrase *Inclusion/exclusion problems*: difficulties in determining what to include or exclude in a word used in an item *Temporal problems*: difficulties in responding to an item if the scale does not fit *Logical problems*: when the item has more than one focus or includes, for example, negations or contradictions *Computational problems*: residual types of problems
Magnitude of the problem's effect on response data *Prominent problems*: when the participants did not understand the content of the item or had insufficient information to answer the item *Minor problems*: when the participants had to reread the item several times and/or asked for help from interviewers but managed to provide a grounded response

Finally, the identified problems were scrutinized in terms of whether the problem was a result of the content of the item or if it was related to the Vietnamese translation of the item.

#### Test–retest survey

The test–retest approach is primarily relevant for instruments assessing constructs that are not expected to change much between two administrations (21). The test survey was conducted in the last week of August 2014, while the retest survey at the CHCs was conducted in the second week of October 2014 and at the district hospital in the second week of December 2014. The time interval between the two administrations (6 and 13 weeks, respectively) was considered long enough for the respondents to have forgotten their previous responses, but short enough to assume that the underlying healthcare context had not changed (22).

In the test–retest survey, we included health providers from all 10 CHCs in one district and from the maternal and neonatal departments at the district hospital. Eligible individuals were full-time providers who had been working for at least 1 year at their current unit. Further, we randomly selected half of the CHWs working for at least 3 years in connection with the included CHCs to participate. These minimum durations of working time were applied to ensure that respondents were well aware of their unit's context. While answering the COACH tool, all respondents from a unit sat in a room together. It was ensured that they could not discuss their answers with their colleagues. A data collection manual was developed to ensure that the COACH tool was introduced in the same manner for all participants. Demographic characteristics of respondents, including age, sex, years after graduation, years working in the current unit, and professional groups, were collected as part of the test survey. Out of 84 eligible respondents, 77 participated in both the test and the retest administration.

For each item, test–retest reliability was analyzed using intraclass correlation coefficient (ICC) with one-way random average measure [ICC (1,*k*)] (23) and percent agreement. The ICC and percent agreement were classified as follows: excellent (>0.80 and >80%), substantial (>0.60–≤0.80 and >60–≤80%), moderate (>0.40–≤0.60 and >40–≤60%), and poor (≤0.40 and ≤40%) (24). In addition, ICC (1,*k*) was computed for each dimension. The systematic differences of dimensions between administrations were tested using the Wilcoxon Rank-Sum Test (25). Further, Bland–Altman plots with 95% limits of agreement (LoA) and coefficient of repeatability were calculated per dimension to explore the size of measurement errors between administrations (26). All analyses were undertaken using R statistical software (25), Psych (27), and MethComp (28) packages.

### Ethical considerations

Ethical approval for this study was obtained from the Provincial Department of Science and Technology in Quang Ninh Province, Vietnam (ref 3934/QDBYT), and the Research Ethics Committee at Uppsala University, Sweden (ref 2005: 319).

## Results

### Think-aloud interviews

In general, the participants found that the COACH tool was clear, well organized, and easy to answer. Most of the items were understood as intended or had minor problems. In total we identified problems with 19 of the 49 items, out of which five items contained prominent problems and 14 items had minor problems. A few items had more than one problem; thus in total we identified 23 problems (ten lexical, five logical, seven inclusion/exclusion, and one computational) (Table 3). Identified problems were evenly distributed across the professions of respondents. In terms of dimensions, we identified problems in the introduction text to two of the eight dimensions. Further, respondents perceived that the content of the *commitment to work, leadership, and informal payment dimensions* could be of a sensitive nature.

**Table 3:** Taxonomy problems, intraclass correlation coefficients, percent agreement, and limits of agreement for items and dimensions of the COACH tool

Dimension	Item	Taxonomy problems^a^	ICC (1,*k*)^b^	Percent agreement	ICC (1,k)^b^	LoA^c^	Lower limit^d^	Upper limit^d^
Organizational resources	1. My unit has enough workers with the right training and skills to do everything that needs to be done.	I/E, minor	0.35	60	0.54	−1.47	−10.38	7.43
	2. My unit has enough workers with the right training and skills to do their job in the best possible way.	–	0.42	62				
	3. My unit has enough space to provide healthcare services.	I/E, minor	0.58	56				
	4. My unit has access to the transport and fuel that are needed to provide healthcare services.	Lex, prominent	0.29	65				
	5. My unit has access to the communication tools (e.g. telephones or radios) that are needed to provide healthcare services.	Lex, prominent	0.56	74				
	6. My unit has enough medicine to provide healthcare services.	Log, minor	0.59	65				
	7. My unit has enough functional equipment, such as a thermometer and blood pressure cuff, to provide healthcare services.	I/E, minor	0.30	65				
	8. My unit has enough disposable medical equipment, such as syringes, gloves, and needles, to provide healthcare services.	–	0.55	69				
	9. If the workload increases, my unit can get additional resources such as medicine and equipment.	I/E, minor	0.59	64				
	10. My unit receives money according to an established financial plan.	Log, minor	0.44	52				
	11. My unit has money that we can decide how to use.	I/E, minor Log, minor	0.63	56				
Community engagement	12. In my unit we ask community members what they think about the healthcare services that we provide	–	0.42	71	0.49	0.1	−4.49	4.51
	13. In my unit we listen to what community members think about the healthcare services we provide.	–	0.34	66				
	14. In my unit we have meetings with community members to discuss health matters.	–	0.32	65				
	15. In my unit we encourage community members to contribute to improving the health of the community.	Lex, minor	0.46	69				
	16. In my unit we encourage other organizations to contribute to improving the health of the community.	Lex, minor I/E, minor	0.45	75				
Monitoring services for action	17. I receive regular updates about my unit's performance based on information/data collected from our unit.	–	0.52	74	0.54	0.18	−4.47	4.84
	18. My unit discusses information/data from our unit in a regular, formal way, such as regularly scheduled meetings.	–	0.54	78				
	19. My unit regularly uses unit information/data to make plans for improving its healthcare services.	–	0.34	69				
	20. My unit regularly monitors its work by comparing it with the unit's action plans.	–	0.54	75				
	21. My unit regularly compares its work with national or other guidelines.	I/E, minor	0.33	58				
Sources of knowledge	22. Clinical practice guidelines.	–	0.64	42	0.72	−0.39	−8.90	8.11
(frequency of use)	23. Other printed material for work (e.g. textbooks, journals).	–	0.26	38				
	24. The Internet.	–	0.89	64				
	25. Electronic decision support (e.g. mobile phone applications or other electronic devices to assist with care and decision-making).	Lex, prominent	0.19	36				
	26. In-service training/workshops/courses.	C, minor	0.63	66				
Commitment to work	27. I am proud to work in this unit.	–	0.58	55	0.61	−0.21	−4.6	3.65
	28. I am satisfied to work in this unit.	–	0.61	62				
	29. I feel encouraged to do my very best at work.	–	0.44	57				
Work culture	30. My unit is willing to use new healthcare practices such as guidelines and recommendations.	–	−0.10	74	0.48	−0.22	−5.41	4.97
	31. My unit helps me to improve and develop my skills.	–	0.35	66				
	32. I am encouraged to seek new information on healthcare practices.	Lex, minor	0.26	68				
	33. My unit works for the good of the clients and puts their needs first.	–	0.52	64				
	34. Members of the unit feel personally responsible for improving healthcare services.	–	0.43	68				
	35. Members of the unit approach clients with respect.	–	0.40	61				
Leadership	36. I trust the unit leader.	–	0.39	65	0.61	−0.19	−5.80	5.41
	37. The leader handles stressful situations calmly.	Lex, minor	0.41	70				
	38. The leader actively listens, acknowledges, and then responds to requests and concerns.	–	0.41	66				
	39. The leader effectively resolves any conflicts that arise.	Lex, minor	0.64	73				
	40. The leader encourages the introduction of new ideas and practices.	–	0.56	66				
	41. The leader makes things happen.	–	0.64	71				
Informal payment	42. Clients must always give informal payment to health workers to access healthcare services.	–	0.32	58	0.16	0.17	−8.16	8.50
	43. Clients are treated more quickly if they make informal payments to health workers.	–	0.44	62				
	44. Medicines or equipment that should be available for free to clients have been sold in my unit.	–	0.55	69				
	45. Health workers are sometimes absent from work earning money at other places.	–	0.39	52				
	46. Health workers in my unit give healthcare services to friends and family first.	–	0.54	55				
	47. Health workers in my unit give jobs or other benefits to friends and family first.	–	0.53	62				
	48. Efforts are made to stop clients from providing informal payment to get appropriate healthcare services.	Lex, prominent Log, prominent	0.50	52				
	49. Efforts are made to stop health workers from asking clients for informal payment.	Lex, prominent Log, prominent	0.07	45				

a Classification according to Conrad and Blair's taxonomy (20): Lex (lexical problems), I/E (inclusion/exclusion problems), Log (logical problems), C (computational problems). Magnitude of the problem's effect on response data: prominent, minor.

b ICC (1,*k*): the intraclass correlation coefficient using the one-way random average measures

c LoA: limits of agreement

d Upper limit = LoA – 1.96×SD; lower limit = LoA + 1.96×SD

### Lexical problems

Lexical problems related to misunderstanding the meaning of words or how words were used. First, despite being familiar with the meaning of single words, some participants could still find it difficult to understand the meaning of items. An example is the dimension of *organizational resources*, where items focusing on different types of resources that the unit ‘has access to’ were misunderstood as resources that were ‘owned by the unit’ (Item 4: *My unit has access to the transport and fuel that are needed to provide healthcare services*). Second, three participants were uncertain of whether the Vietnamese translation of ‘encourage’ meant ‘being counseled’ or ‘being supported’ to do something. When the respondents encountered these difficulties, the interviewer could explain the meaning of the items, after which some participants suggested changes in phrasing to address the lexical problems. Out of the 10 items identified as having lexical problems, five were judged to have prominent problems.

### Inclusion and exclusion problems

The main problems in this category related to problems of exclusion, where a lack of examples to assist respondents in determining whether concept(s) were within the content of the items was highlighted. Thus, for clarification, participants suggested adding examples to items. An example was that one participant understood the word ‘equipment’ to imply ‘low-tech equipment’ that should be available at CHCs (Item 7: *My unit has enough functional equipment, such as a thermometer and blood pressure cuff, to provide healthcare services*). However, the Ministry of Health in Vietnam considers an ultrasound machine as a standard device in CHCs (29). Despite the lack of an ultrasound machine at her unit, the participant rated the level of agreement as *agree* regarding having enough functional equipment because she perceived that her unit had enough ‘low-tech equipment’. All seven items with inclusion/exclusion problems were judged to be minor problems.

### Logical problems

The main logical problem was false presuppositions. One such example was that CHWs and CHC staff had difficulties in rating their level of agreement with items regarding the availability of financial resources in their unit (in the *organizational resources* dimension), as it was only the head of the CHC who was considered to have that type of information. Another logical problem was the reluctance of respondents to rate their agreement to the last two items of the COACH tool due to the reversed order of meaning of the items (having positive implications) compared with the other items in the dimension (having negative implications). Out of the identified five items with logical problems, two were judged as prominent problems.

### Computational problems

One computational problem was the difficulty in choosing the frequency of using a certain *source of knowledge* in what was defined as a ‘normal’ month. This was judged as a minor problem. Further, participants brought up the risk of not providing sincere answers to topics perceived as sensitive, including items in the *commitment to work*, *leadership* and *informal payment* dimensions. Participants noted that future respondents might not provide truthful responses or might refuse to answer items relating, for example, to whether their units were engaged in informal payment or on how they perceived the leadership under which they worked.

### Test–retest survey

A total of 77 respondents in both the test and retest administrations were evenly distributed into three professional groups. Most of the respondents were women (78%). Their mean age was 41 years and their mean years of working in the current unit was 3.1 years. Responses to the COACH tool were not equally distributed, as a majority (75%) rated the items as either *agree* or *strongly agree*. There were only 11 missing responses; thus, no imputations were undertaken.

#### Test–retest reliability for dimensions

The results of the test–retest are presented in Table 3 and Supplementary file 1. All dimensions except *informal payment* (ICC=0.16) had an ICC value ranging from 0.5 to 0.7, which demonstrated a moderate to substantial agreement. The negative LoA in five out of eight dimensions indicated that the test scorings in most cases were lower than the retest scorings. Wilcoxon Signed-Rank tests, however, only showed significant difference between the two survey administrations for the *organizational resources* dimension.

**Fig. 1:**
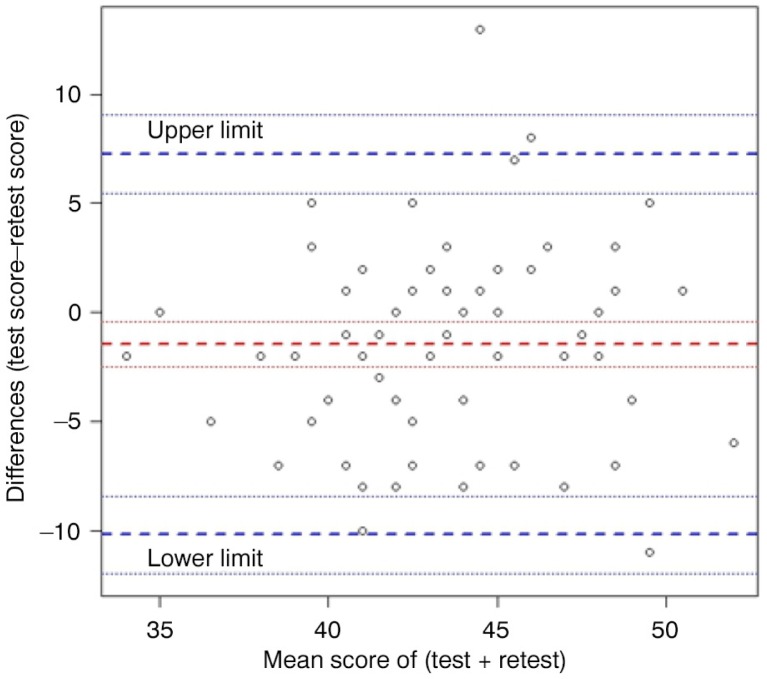
Bland–Altman plot of *organizational resources* dimension.

Figure 1 exemplifies a Bland–Altman plot displaying the *organizational resources* dimension, showing the score difference (*y*-axis) against the mean (*x*-axis) between the two administrations. The range between the lower limit and the upper limit of agreement (about 18) was wide and the data points were dispersed across the zero-difference line (*y*-axis).

#### Test–retest reliability for items

The ICC values and percent agreement per item are presented in Table 3. About one-third of the items had poor ICC values (≤0.40), whereas the remaining had moderate to substantial ICC values (>0.40). One item had an excellent ICC value (>0.80). The four dimensions having the highest proportion of items classified as having *poor* ICC values (≤0.40) were *work culture* (67%), *community engagement* (40%), *monitoring services for action* (40%), and *sources of knowledge* (40%). The remaining four dimensions, having the highest proportion of items classified as *moderate* (>0.40), were *commitment to work* (100%), *leadership* (83%), *organizational resources* (73%), and *informal payment* (63%). In terms of percent agreement, almost all the items (96%) had moderate to substantial agreement (>40%) (Table 3). Further, 7 out of 19 items with a low ICC value (≤0.40) also comprised taxonomy problems. Two of the three items with the lowest ICC values (<0.20) had lexical and computational problems that were judged as prominent problems.

## Discussion

Overall, our findings suggest that the COACH tool was understood as intended and reliable for measuring aspects of healthcare context perceived to be important for KT. The tool, however, comprised seven prominent problems relating to some items and had three dimensions with items perceived to be of a sensitive nature. In the test–retest, two-thirds of items and seven of eight dimensions were found to have a moderate to substantial agreement between survey administrations, demonstrating that the instrument has reasonable stability.

### Think-aloud interviews

Lexical problems were the most common problems in the interviews, and they also accounted for the highest number of prominent problems (five out of seven). Despite a careful translation of the COACH tool (13, 30), four of these five problems appeared to be attributable to the translation of the tool into Vietnamese. As a result, our findings indicate that there is a need to review the translation of these items. Rephrasing ambiguous wording and providing generic examples that clarify the content of the item might help to address some of these problems.

Three dimensions, *leadership*, *informal payment*, and *commitment to work*, contained items that respondents perceived to be of a sensitive nature. Collecting data that accurately reflect respondents’ thoughts about sensitive issues is difficult (31), partly due to the fear of repercussions, which could influence their answers (32). Informal payment is a particularly sensitive issue and has been recognized to be difficult to measure, especially in LMICs (33, 34). Respondents might provide socially acceptable answers to avoid embarrassment for themselves or to please their leaders or the researchers conducting the survey (35). From our think-aloud interviews, participants suggested that confidentiality and anonymity should be further stressed as part of the introduction to the COACH tool. Anonymity, confidentiality, and using a non-judgmental tone have been suggested to increase the opportunities to receive sincere answers from respondents (36, 37). When using the COACH tool in the future, it is thus important to strive for confidentiality, for example, through having each respondent filling in the tool in a secluded area, instead of in a room together with several colleagues or by collecting data by other means (38).

Misunderstanding or not reading the introduction as intended (lexical and computational problems) was a common problem within the *sources of knowledge* dimension. This problem is grave as the instruction contains important information, such as time frame, which needs to be carefully considered while rating the level of agreement (39). To overcome this problem in future use, it might be necessary to carefully introduce the tool, including underlining the importance of carefully reading the introduction and of asking for help if specific parts are difficult to understand.

Another difficulty detected in the think-aloud interviews was the lack of information needed for respondents to be able to provide answers to what was being asked (logical problems). This problem was particularly obvious for CHWs, who, for example, lacked knowledge about the financial situation at the CHC. This point might reflect a potential difficulty using the COACH tool with CHWs in Vietnam, as they only work part-time as health providers and are mostly active outside the CHC. In the development of the COACH tool, the CHWs in Vietnam also had lower reliability scores compared with CHWs in the other four countries (Bangladesh, South Africa, Nicaragua, and Uganda) where development tests were undertaken (13). This difference might be attributed to the difference between the roles of CHWs in Vietnam and other settings. An option to address this problem might be to exclude items that are not relevant to a specific group.

### Test–retest survey

The moderate to substantial ICC values in most of the dimensions demonstrated the acceptable stability of the responses received in repeated applications of the COACH tool. The moderate ICCs were also illustrated by the small LoA of the dimensions but a wide range between the lower and upper LoA and dispersed data points between test and retest. Our test–retest reliability findings are similar to the psychometric evaluations of other tools measuring organizational structures and working climate (40, 41) and also similar to the characteristics of an instrument for evaluating the implementation of clinical practice guidelines (42). All three studies presented ICC values ranging between 0.5 and 0.7. In term of items, two-thirds of items had moderate to substantial ICC values, whereas almost all of the items had moderate to substantial percent agreement. This finding is consistent with the criticism of the overestimation of the level of agreement by only using percent agreement (43, 44). A potential explanation for having relatively many items with low ICC values is the high proportion of ratings with right-side skewed responses (*agree*/*strongly agree*), indicating relatively homogeneous scorings in the test–retest survey (43). Further, more than one-third of the items with low ICC had taxonomy problems, and two out of the three items with the lowest ICC values had prominent taxonomy problems. These findings underline that think-aloud interviews can be a helpful method to revise and improve items in the COACH tool.

The *informal payment* dimension had one item with exceptionally low ICC (0.07), which in turn led to the dimension having the lowest ICC (0.16). Furthermore, the mix of items in this dimension, alternating between positively and negatively posed questions, was emphasized as problematic in the think-aloud interviews and might have contributed to the low ICC of these items. Despite the fact that informal payment is repeatedly brought up as a major obstacle to the quality of health services in LMICs (33, 45), such a component is not common in tools assessing the healthcare context (46). Therefore, additional studies are needed to examine the validity and reliability of this dimension.

### Methodological considerations

To address subjectivity, a potential flaw when analyzing think-aloud interviews, we opted to use the Conrad and Blair taxonomy, a structured framework intended to increase objectivity in the analysis (47). Moreover, all of the authors discussed the identified problems to achieve consensus in the analysis and synthesis. In terms of the test–retest survey, the results in this study were strengthened by having very few missing responses. The difference of time intervals between CHCs and district hospital in the test–retest (6 and 14 weeks, respectively) might have influenced the findings. However, the trait that the COACH tool measures, healthcare context, is believed to be a stable construct over a short time period (22), which was about 3.5 months at the longest in our study. Moreover, some specific aspects of context have been reported as relatively stable over time, including commitment to work (40) and leadership (48). Other studies focusing on organizational culture and work climate have reported a stable measurement of constructs, even when having a longer time interval between the test and the retest administration (40, 49).

## Conclusions

The think-aloud interviews showed that the items in the COACH tool, in general, were clear and easy to answer. The test–retest demonstrated that the instrument has an acceptable level of stability. Thus, the main parts of the translated version of the COACH tool appear to be relevant for use among different types of healthcare provider groups in Vietnam. There is, however, a need to revisit the items comprising translation problems and low ICC values. To avoid ambiguous wording, some items will be rephrased; in addition, generic examples will be provided for clarification. The findings also indicate that some items might not be relevant for CHWs in general and for CHWs in Vietnam in particular. Moreover, future users of the COACH tool should ensure that respondents can complete it in private to ensure confidentiality and to acquire the most trustworthy responses possible.

## Supplementary Material

Response process and test–retest reliability of the Context Assessment for Community Health tool in Vietnam

## Paper context

Improved understanding of local health care context is an important aspect of supporting the implementation of evidence-based practices. The recently developed Context Assessment for Community Health (COACH) tool, a tool particularly developed for measuring aspects of local health care context, is a promising way to investigate context. However, there is a need to generate further evidence to establish the reliability and validity of the tool. This study aims to explore the understanding of the Vietnamese translation of the COACH tool among health providers in Vietnam (response process) and establish the stability of the tool over time (test-retest). Our results suggest that the Vietnamese translation of the COACH tool is generally perceived to be clear and easy to understand and has acceptable stability. The tool, thus, is useful to measure aspects of health care context in relation to knowledge translation in Vietnam.
