# Supplementary material for: Response process and test–retest reliability of the Context Assessment for Community Health tool in Vietnam
Source: Glob Health Action. 2016 Jun 10;9:10.3402/gha.v9.31572. doi: 10.3402/gha.v9.31572 (PMC4904068; doi:10.3402/gha.v9.31572)
Supplement: Response process and test–retest reliability of the Context Assessment for Community Health tool in Vietnam [file GHA-9-31572-s001.doc]

## Additional file 1 – Descriptive information of the dimensions of the COACH tool

|  | **Test/ Retest** | **No of missing** | **No of items** | **Mean (SD)** | **Median** | **Min** | **Max** |
| --- | --- | --- | --- | --- | --- | --- | --- |
| Organizational resources | Test | 3 | 11 | 42.7 (4.3) | 42.5 | 33 | 52 |
|  | Retest | 0 |  | 44.1 (3.9) | 44 | 35 | 55 |
| Community engagement | Test | 0 | 5 | 21.1 (1.9) | 20 | 15 | 25 |
|  | Retest | 0 |  | 21.1 (2.0) | 20 | 14 | 25 |
| Monitoring services for action | Test | 0 |  | 20.8 (2.1) | 20 | 14 | 25 |
|  | Retest | 0 | 5 | 20.6 (2.1) | 20 | 14 | 25 |
| Sources of knowledge | Test | 1 |  | 19.5 (4.6) | 20 | 11 | 28 |
|  | Retest | 0 | 5 | 19.8 (4.5) | 20 | 13 | 30 |
| Commitment to work | Test | 0 |  | 12.7 (2.0) | 13 | 8 | 15 |
|  | Retest | 0 | 3 | 12.9 (1.7) | 12 | 6 | 15 |
| Work culture | Test | 1 |  | 25.6 (2.0) | 25 | 23 | 30 |
|  | Retest | 0 | 6 | 25.8 (2.4) | 25 | 18 | 30 |
| Leadership | Test | 0 |  | 24.8 (2.4) | 24 | 18 | 30 |
|  | Retest | 0 | 6 | 25.0 (2.9) | 24 | 16 | 30 |
| Informal payment | Test | 1 |  | 17.1 (3.4) | 16 | 8 | 29 |
|  | Retest | 1 | 8 | 16.9 (2.7) | 16 | 11 | 24 |

**Note:** The mean values between dimensions are not comparable due to them having different numbers of items.
